# GSK3β and Tau Protein in Alzheimer’s Disease and Epilepsy

**DOI:** 10.3389/fncel.2020.00019

**Published:** 2020-03-17

**Authors:** Danira Toral-Rios, Pavel S. Pichardo-Rojas, Mario Alonso-Vanegas, Victoria Campos-Peña

**Affiliations:** ^1^Departamento de Fisiología Biofísica y Neurociencias, Centro de Investigación y de Estudios Avanzados del IPN, Mexico City, Mexico; ^2^Facultad de Ciencias de la Salud, Universidad Autónoma de Baja California, Ensenada, Mexico; ^3^Centro Internacional de Cirug#x000ED;a de Epilepsia, Instituto Nacional de Neurología y Neurocirugía, HMG, Hospital Coyoacán, Mexico City, Mexico; ^4^Laboratorio Experimental de Enfermedades Neurodegenerativas, Instituto Nacional de Neurología y Neurocirugía, Mexico City, Mexico

**Keywords:** tau protein, GSK3β, epilepsy, neurodegeneration, hippocampal sclerosis

## Abstract

Alzheimer’s disease (AD) is the most common form of dementia present in older adults; its etiology involves genetic and environmental factors. In recent years, epidemiological studies have shown a correlation between AD and chronic epilepsy since a considerable number of patients with AD may present seizures later on. Although the pathophysiology of seizures in AD is not completely understood, it could represent the result of several molecular mechanisms linked to amyloid beta-peptide (Aβ) accumulation and the hyperphosphorylation of tau protein, which may induce an imbalance in the release and recapture of excitatory and inhibitory neurotransmitters, structural alterations of the neuronal cytoskeleton, synaptic loss, and neuroinflammation. These changes could favor the recurrent development of hypersynchronous discharges and epileptogenesis, which, in a chronic state, favor the neurodegenerative process and influence the cognitive decline observed in AD. Supporting this correlation, histopathological studies in the brain tissue of temporal lobe epilepsy (TLE) patients have revealed the presence of Aβ deposits and the accumulation of tau protein in the neurofibrillary tangles (NFTs), accompanied by an increase of glycogen synthase kinase-3 beta (GSK3β) activity that may lead to an imminent alteration in posttranslational modifications of some microtubule-associated proteins (MAPs), mainly tau. The present review is focused on understanding the pathological aspects of GSK3β and tau in the development of TLE and AD.

## Introduction

Alzheimer’s disease (AD) is characterized by progressive memory loss, behavioral changes, and cognitive decline. Histopathologically, it is defined by the presence of neuritic plaques (NPs) conformed by fibrillar accumulation of amyloid beta-peptide (Aβ) and neurofibrillary tangles (NFTs) formed by the microtubule-associated protein (MAP) tau. These lesions produce microglial and astrocyte activation, leading to synaptic loss and neuronal death (Meraz-Ríos et al., 2013). It has been proposed that Aβ can interact with Frizzled receptors, impairing the Wnt/β-catenin signaling pathway conducting to increased activity of the main tau protein kinase, glycogen synthase kinase-3 beta (GSK3β). Tau hyperphosphorylation promotes the destabilization of cytoskeleton microtubules (MTs), leading to axonal transport abnormalities and neuronal death (Inestrosa and Toledo, 2008). Aβ and tau protein have been linked to excitotoxicity and hyperexcitability (Holth et al., 2013). The relationship of tau and excitability is important in view of the increased incidence of unprovoked seizures observed in AD patients, which may result from alterations in neural circuits and neurodegenerative processes (Pandis and Scarmeas, 2012). In this way, seizures and hyperexcitability of the network could contribute to accelerating the development of cognitive impairment. In addition, the neuronal circuits affected in AD involve brain areas that have been shown to be highly epileptogenic and are strongly related to temporal lobe epilepsy (TLE), the most common type of epilepsy. Histopathological studies in TLE patients have shown the presence of hyperphosphorylated tau protein aggregates (Sen et al., 2007; Thom et al., 2011; Tai et al., 2016). The present review aims at assessing the role of tau and GSK3β proteins in the development of AD and TLE.

## Tau Protein

**Figure 1:**
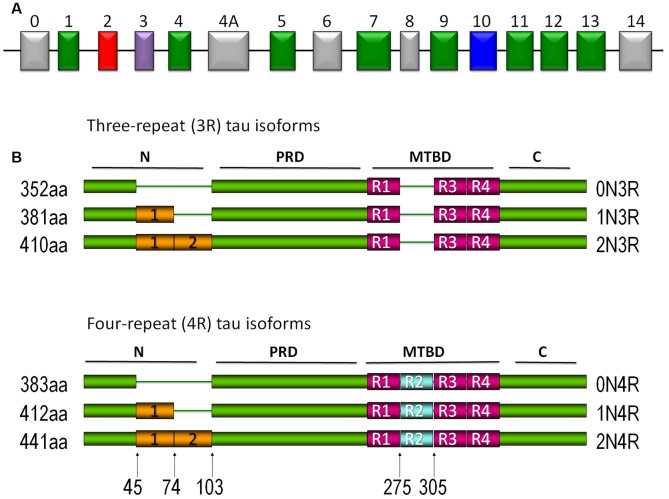
Tau protein. **(A)** Tau gene. Exons 2, 3, and 10 are alternatively spliced in the central nervous system (CNS). Exons 9–12 each contain the microtubule-binding domain (MBD). Exons 4a and 6 have been expressed in isoforms of the peripheral nervous system, whereas exon 8 has not been reported in any isoform. **(B)** Different isoforms of tau protein are expressed in the CNS. The expression of different isoforms is regulated by development. Isoforms with three repeated domains are expressed preferentially in fetal stages, whereas in the adult stages they are characterized by the presence of the six isoforms. The repeated domains that bind to microtubules (MTs) are designated as *R1*, *R2*, *R3*, and *R4*. Another characteristic is the presence or absence (*0N*) of one (*1N*) or two (*2N*) inserts located in the amino terminus of the protein.

Tau is a type II MAP, mainly located in axons. It is a heat-stable protein that prevents MT assembly and stabilization in the central nervous system (CNS). Human tau is encoded by the *MAPT* gene located on chromosome 17q21 and consists of 16 exons (Andreadis et al., 1992; Andreadis, 2005; Figure 1A). Six different isoforms of the protein are expressed in the adult human brain. Each isoform contains three or four microtubule binding repeats (3R/4R) and the presence or absence of one or two N-terminal inserts (Buée et al., 2000; Martin et al., 2011; Figure 1B).

Under normal conditions, tau interacts with motor proteins such as dynein and kinesin, participating in retrograde and anterograde transport (Dixit et al., 2008), in embryonic development, long-term potentiation (LTP; Ahmed et al., 2014), and long-term depression (LTD; Kimura et al., 2014; Regan et al., 2015). Under pathological conditions, it self-assembles into insoluble structures, known as paired helical filaments (PHFs; Goedert, 1999). Two tau posttranslational modifications are present in PHFs: hyperphosphorylation and truncation (Flament et al., 1990; Alonso et al., 1996; Hasegawa et al., 1998).

Hyperphosphorylation prevents tau microtubule binding, resulting in an altered cytoskeletal stability (Evans et al., 2000), a subsequent loss of axonal transport, and other signaling-related functions (Mandelkow et al., 1995); it has also been considered the primary event that triggers the tau pathological aggregation in filaments (Grundke-Iqbal et al., 1986; Wood et al., 1986; Alonso et al., 1996).

## Tau and Epilepsy

In recent years, tau protein has been implicated in the disruption of neuronal synchronization and hyperexcitability; in this way, it could also be linked to epilepsy. Even though the specific pathologic mechanisms are yet to be clarified, there are different reports supporting these claims.

Some models of tau pathology have been shown to induce drastic changes in connectivity and strong uncoupling of the gamma–theta oscillations. However, no signs of epileptiform activity were registered (Ahnaou et al., 2017). Challenging this idea, a transgenic mouse model of human amyloid precursor protein (hAPP) presented an overproduction of Aβ and consequent development of spontaneous seizures. Adding a tau gene knockout to this model revealed that tau reduced levels, prevented *N*-methyl-D-aspartate receptor (NMDAR) dysfunction, impaired LTP, ameliorated cognitive decline, and reduced epileptiform activity in the hippocampus (Roberson et al., 2007, 2011).

Another interesting study which evaluated the relationship of tau with hyperexcitability is the *Kcna1*^−/−^ mouse, a TLE model. These mice have a null allele for the alpha subunit of Kv1.1, a voltage-gated potassium channel that conditions the development of spontaneous seizures in the third week of life. The tau gene knockout in this model reduced the occurrence and frequency of spontaneous seizures and promoted survival, specifically in the CA3 pyramidal region of the hippocampus (Holth et al., 2013). Another similar model demonstrated that Aβ-induced hyperexcitability was associated with a reduction in the levels of Kv4.2, a dendritic potassium channel important in regulating dendritic excitability and synaptic plasticity. Interestingly, enough of both of these phenomena were dramatically reduced in tau knockout mice, suggesting that tau may have a direct role in modulating neuronal excitability (Hall et al., 2015). Similar results were observed in a Tau^−/−^ mouse model treated with Pentylenetetrazole (PTZ). In this case, it was observed that a reduction in tau expression protects against seizure severity (DeVos et al., 2013). This is interesting considering that it is possible that endogenous tau levels in AD patients may influence the risk of developing seizures.

Although having higher basal levels of tau in the cerebrospinal fluid does not cause seizures in itself, these patients are more likely to develop seizures after an injury, for example, after an ischemic stroke or traumatic brain injury (Camilo and Goldstein, 2004; Kwan, 2010). For this reason, in the future, it could be important to identify high-risk patients through genetic analysis, maybe by identifying polymorphisms in the tau gene (Myers et al., 2007; Kauwe et al., 2008) or levels of tau in the cerebrospinal fluid (Cruchaga et al., 2013), to determine whether, in this population, it may be useful to provide prophylactic antiepileptic therapy.

Hyperphosphorylated tau aggregates and NFTs have been observed in several patients with epilepsy (Sen et al., 2007; Thom et al., 2011; Tai et al., 2016). Moreover, a study on temporal lobe resections in patients with refractory epilepsy demonstrated that the presence of hyperphosphorylated tau and its accumulation in pre-tangles and NFTs seem to correlate with cognitive decline (Tai et al., 2016). Evidence suggests that prolonged or recurrent seizures in epilepsy patients can cause or exacerbate cognitive impairment (Holmes, 2015; Kneynsberg et al., 2017). Pathologic tau has been correlated with excitotoxicity (Roberson et al., 2007), epilepsy (DeVos et al., 2013), and cognitive impairment, particularly memory (Holmes, 2015). However, to date, the specific mechanisms explaining how hyperphosphorylated tau induces hyperexcitability are an area of active research.

Epileptogenesis is associated with an imbalance between excitatory and inhibitory neurotransmitters. The principal excitatory neurotransmitter dysregulated in epilepsy is glutamate, which favors excitotoxicity by the overstimulation of NMDAR. Furthermore, the activation of NMDAR has been proposed as another mechanism promoting tau phosphorylation. Through this process, tau could regulate NMDAR activation, synaptic plasticity, and neurotoxicity (Mondragón-Rodríguez et al., 2012).

These potential roles of tau opened a new field of study to establish a direct relationship with neuropathology and knowledge of other diseases such as TLE, which courses with seizures linked to imbalances of the hippocampal neuronal networks and neurodegenerative processes. In this way, further research must be done to determine the exact mechanisms of how tau plays a pivotal role in epileptogenesis and, therefore, make potential interventions of diagnostic, therapeutic, and prognostic value.

## GSK3

GSK3 is a proline-directed kinase; there are two isoforms, glycogen synthase kinase-3 alpha (GSK3β) and beta (GSK3β) encoded by chromosomes 19 and 3, respectively. GSK3β is mainly expressed in the CNS, normally located in axons, and is the main kinase that phosphorylates tau protein (Muyllaert et al., 2008). GSK3β could be inactivated through phosphorylation in serine 9 and activated by phosphorylation in tyrosine 216. GSK3β inactivation is regulated by the PI3K/Akt and Wnt/β-catenin pathways. In AD, both are altered and favor an increased activity of GSK3β (Hooper et al., 2008).

**Figure 2:**
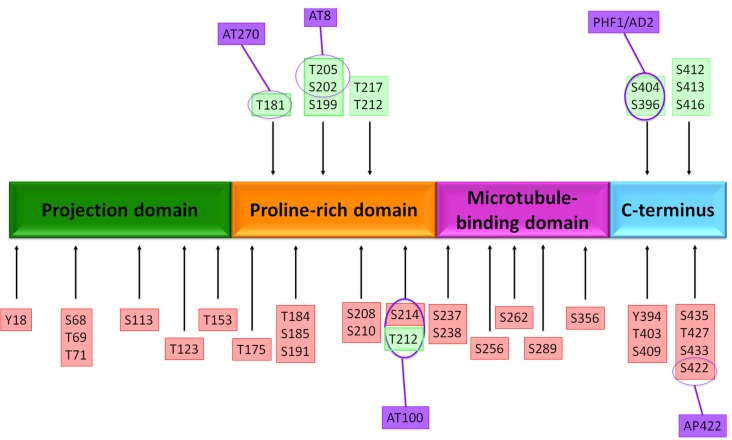
Putative phosphorylation sites on tau protein. The native tau protein is phosphorylated in serine or threonine residues and contains 85 putative phosphorylation sites. Several amino acids are phosphorylated in Alzheimer’s disease (AD) brain (*red boxes*) and both normal and AD brains (*green boxes*). The localization of antibody epitopes is indicated (*purple circles*).

Overexpression or overactivity of GSK3β increases tau phosphorylation, allowing it to disassemble from MTs, leading to axonal transport alterations, hippocampal neurodegeneration (Avila et al., 2006; Muyllaert et al., 2008), and learning impairment (Gómez De Barreda et al., 2010). GSK3 is able to phosphorylate tau at 42 sites (Figure 2; Hanger et al., 2009; Martin et al., 2013). Also, GSK3 activity correlates with the amount of NFTs in AD brains (Leroy et al., 2002). Another implication of the kinase in AD is the modulation of the intrinsic pathways of cellular apoptosis which could be favored by the Aβ peptide (Takashima et al., 1996). GSK3β promotes Bax activity, a pro-apoptotic protein, and inhibits transcription factors that avoid cell susceptibility to toxic insults. One of these factors is the heat shock transcription factor 1 (HSF1), which modulates responses to cellular stress (Mines et al., 2011).

Recent studies have shown that GSK3β is related to memory loss and learning impairment; activation and overexpression of the kinase decrease LTP and increase LTD through the modulation of NMDA receptors (Peineau et al., 2007; Salcedo-Tello et al., 2011). Inhibition of GSK3β by lithium protects cells against neurodegeneration and significantly reduces phospho-tau levels and the spatial learning and memory deficits in a splenectomized rat model (Tan et al., 2010).

## GSK3β and Epilepsy

GSK3β could be linked to hyperexcitability and epileptogenesis due to its physiological roles in synaptic plasticity modulation and the control of neuron cytoskeleton dynamic. In a rat model, seizures and hippocampal neuronal damage induced with kainic acid (KA) could stimulate GSK3β activity, inhibiting the Wnt/β-catenin pathway. Administration of lithium, a GSK3β inhibitor, in these rats prevented hippocampal neuronal damage, but not KA-induced seizures (Busceti et al., 2007). Dysfunctional neurogenesis is frequently observed in the latter stages of TLE and has been associated with Wnt/β-catenin pathway inhibition. GSK3β inhibitors restore the pathway and recover neurogenesis (Huang et al., 2015); furthermore, GSK3β activity is also regulated by the PI3k/Akt pathway. This pathway resulted as impaired in KA and electroconvulsive epilepsy models and also could promote GSK3β activation, neuronal damage, and tau phosphorylation before seizures (Crespo-Biel et al., 2007; Gangarossa et al., 2015).

In line with the above information, other studies evidenced a pro-apoptotic trend in Bcl2/Bax expression in a Western blot assay 48 h after KA injection; Bcl2 family proteins seem to be an important downstream target of GSK3β (Linseman et al., 2004). In this regard, decreased Bcl2 expression may be another consequence of the neurodegenerative process (Bhowmik et al., 2015). All of these findings suggest that alterations of GSK3β after an excitotoxic insult play a key role not only in neurodegeneration but also in the disruption of the normal neuronal survival signaling pathways.

Even though GSK3β has not been demonstrated to produce epilepsy in itself, there is evidence showing that GSK3β inhibition does result in anticonvulsant activity, even after brain excitability has been altered from normal, healthy conditions into a “pro-epileptic” state (Aourz et al., 2019). Furthermore, GSK3β alterations do increase seizure susceptibility, and increased GSK3β activity has been evidenced in other epilepsy models (Lohi et al., 2005; Tripathi et al., 2010; Lee et al., 2012).

Despite the increased GSK3β activity having been probed in other epilepsy models (Lohi et al., 2005; Tripathi et al., 2010; Lee et al., 2012), the addition of KA into hippocampal slices of mice that overexpressed a constitutively active form of GSK3β produced a decreased progression of induced epileptogenesis, probably mediated by the reduced phosphorylation of the GluA1 subunit of the glutamate α-amino-3-hydroxy-5-methyl-4-isoxazolepropionic acid (AMPA) receptor (Urbanska et al., 2019). This result seems contradictory because of the beneficial effects attributed to GSK3β inhibition, but a recent study exposed that the brain has limited tolerance for the modulation of GSK3β activity on hippocampal damage related to the severity of status epilepticus (Engel et al., 2018).

Another strong hypothesis of GSK3β and its role in epileptogenesis favoring TLE is the close relation with NMDAR and its stimulation, which may induce excitability in the acute stages of the disease (Liu et al., 2017).

**Figure 3:**
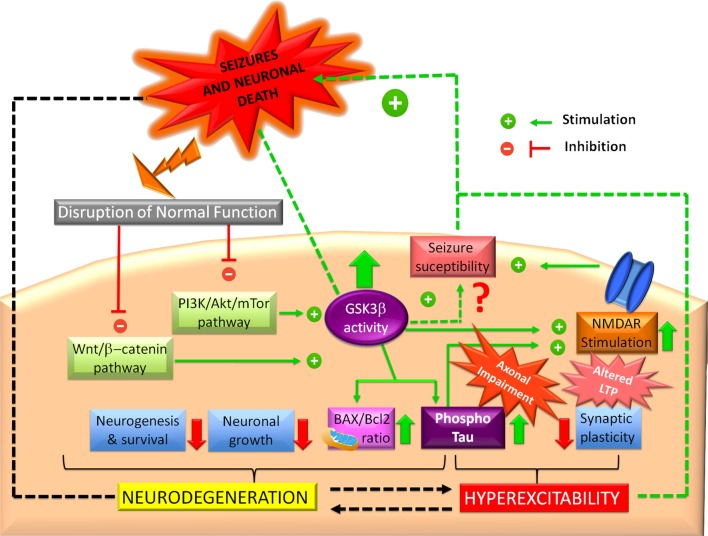
Glycogen synthase kinase-3 beta (GSK3β and epilepsy. Impaired PI3K/Akt and Wnt/b-catenin signaling pathways can induce GSK3β increased activity, which could promote *N*-methyl-D-aspartate receptor (NMDAR) overstimulation, leading to hyperexcitability and synaptic plasticity alterations. Probably, this mechanism represents the major link between GSK3β and seizures in the hippocampus, although some studies have proposed that GSK3β increased activity confers protection. In another way, GSK3β could favor tau hyperphosphorylation, promoting axonal transport impairment and hippocampal neuronal death. Hyperphosphorylated tau actively participates in hyperexcitability and neurodegeneration. GSK3β also promotes a mitochondrial pro-apoptotic profile (high BAX/Bcl2 ratio). In this sense, GSK3β also could trigger dysfunctional neurogenesis, which prevents recovery of the synaptic equilibrium of the hippocampus, one neurogenic structure.

All the evidence mentioned above remark on the importance of GSK3β homeostasis in preventing epilepsy. Even though an excitotoxic insult may initially disrupt GSK3β, once this happens, GSK3β actively plays a role in increasing seizure susceptibility (Figure 3). There is no doubt that GSK3β alterations promote the disruption of normal survival signaling pathways and hyperexcitability, which, together, could produce epileptogenesis and neurodegeneration.

## Therapeutics

As previously mentioned, tau and GSK3β have been associated with several neurological disorders, including AD and TLE; therefore, both represent potential therapeutic targets.

Interestingly, the efficacy of various antiepileptic drugs (AEDs) has been tested in epilepsy experimental models and in AD patients, showing a promising function in cognitive impairment prevention (Sánchez et al., 2018). Since tau hyperphosphorylation is the main mechanism responsible for NFT formation, it has been suggested that inhibiting different tau kinases such as CDK5 and GSK3β, involved in tau hyperphosphorylation, could reduce their aggregation (Xie et al., 2017; Holzer et al., 2018), observed in AD (Morris et al., 2011) and epilepsy (Sen et al., 2007; Thom et al., 2011; Tai et al., 2016).

When AEDs are used in healthy volunteers and different neuropsychological variables are measured, researchers have found that, in general, AEDs like carbamazepine, phenytoin, and valproate have mild cognitive effects (Meador et al., 1995; Martin et al., 2001; Salinsky et al., 2002). In contrast, when the cognitive effects of oxcarbazepine, gabapentin, and carbamazepine were evaluated in healthy volunteers, the results showed that individuals presented a better performance in the focused attention task and manual writing speed, without any effect on long-term memory (Curran and Java, 1993; Meador et al., 1999). It is worth mentioning that older patients are generally more sensitive to the negative cognitive effects of some AEDs due to the complex interactions among several factors (neuropathologies, genetics, the effects of seizures, psychosocial background, etc.; Kwan and Brodie, 2001).

The efficacy of AEDs in preventing seizures in AD patients has been tested. However, reports regarding their cognitive effects in the elderly are relatively limited, specifically in the elderly with AD. Most of the old AEDs exert negative effects on mood, behavior, and cognitive functions, such as memory (Craig and Tallis, 1994; Meinhold et al., 2005). In contrast, newer AEDs appear to be less harmful and may even produce a slight cognitive benefit, even though some of these newer drugs like topiramate appear to impair language and verbal memory (Fritz et al., 2005). Other various new AEDs like lamotrigine and gabapentin do not impair memory and may have slight beneficial effects on memory, and even mood (Tekin et al., 1998; Meador et al., 1999). Levetiracetam has demonstrated cognitive benefits, which can be measured in routine clinical practice, particularly in attention and oral fluency (Cumbo and Ligori, 2010).

The main objective of AED therapy is to improve the patient’s quality of life, and even though the main aim is to reduce the quantity of seizures, the current review may provide additional information, which may aid physicians and their patients in making treatment decisions when considering other therapeutic variables like cognition.

The most advanced protein kinase inhibition strategy in the clinic so far has been aimed at GSK3β, leading to the development of novel drugs such as Tideglusib (Hooper et al., 2008; Medina, 2018). Treatment with this thiadiazolidinone (TDZD) compound resulted in decreased amyloid deposition, lower levels of tau phosphorylation, and prevention of memory deficits in a transgenic mouse model (APP^sw^-tau^vlw^; Serenó et al., 2009). Lithium and valproic acid have been used in epilepsy for their anticonvulsant activity and in other psychiatric disorders (Jope and Roh, 2006). Moreover, these inhibitors and TDZD-8, a selective inhibitor of GSK3β, promote the phosphorylation of serine 9, which conducts to GSK3β inactivation (Zhang et al., 2003). The inhibition strategy reduces tau hyperphosphorylation, prevents neuronal death, and inhibits the alteration of neurogenesis processes in the hippocampus in the setting of status epilepticus (Busceti et al., 2007; Bhowmik et al., 2015; Huang et al., 2015; Urbanska et al., 2019). Memantine and ifendropil are NMDAR antagonists and have been demonstrated to conduct to GSK3β inactivation and reduction of tau phosphorylation (Liu et al., 2017). Recently, the use of potent and selective GSK3β inhibitors such as indirubin and BIO-acetoxime showed anticonvulsant properties (Aourz et al., 2019). In this report, PTZ-treated zebrafish larvae were treated with both compounds, which exhibited anticonvulsant activity, with a reduction of epileptiform discharges. In the same way, indirubin and BIO-acetoxime showed anticonvulsant activity in the 6-Hz refractory seizure mouse model (Aourz et al., 2019).

Acetylation is another posttranslational modification that leads to impaired tau function and promotes its pathological aggregation; hence, tau acetylation inhibitors are proposed as a potential therapeutic strategy for AD and other tauopathies (Medina et al., 2016). Salsalate is a nonsteroidal anti-inflammatory drug which inhibits acetyltransferase p300-induced tau acetylation; this drug has been shown to rescue tau-induced memory deficits and prevent hippocampal atrophy in a mouse model of frontotemporal dementia (FTD; Min et al., 2015). Leuco-methylthioninium bis-hydromethanesulfonate (LMTM) is currently in phase III clinical trials and has shown efficacy in inhibiting tau aggregation (Bulic et al., 2013). Passive and active tau immunotherapy is a promising strategy which has been designed for clearing toxic tau species such as monomers, oligomers, prefilaments, granules, fibrils, or insoluble aggregates present in tauopathies and related disorders. The main objectives of immunotherapy are to reduce tau aggregation, prevent neurodegeneration, and improve cognitive functions (Bittar et al., 2018; Medina, 2018; Novak et al., 2018).

Even though all of these therapeutic strategies mainly target AD, it is clear that, in the future, they may well serve for the treatment of other tauopathies, including TLE. Hence, it is important to procure and encourage the potential therapeutic efficacy of tau and GSK3β-based strategies in the future of these diseases.

## Conclusions

**Figure 4:**
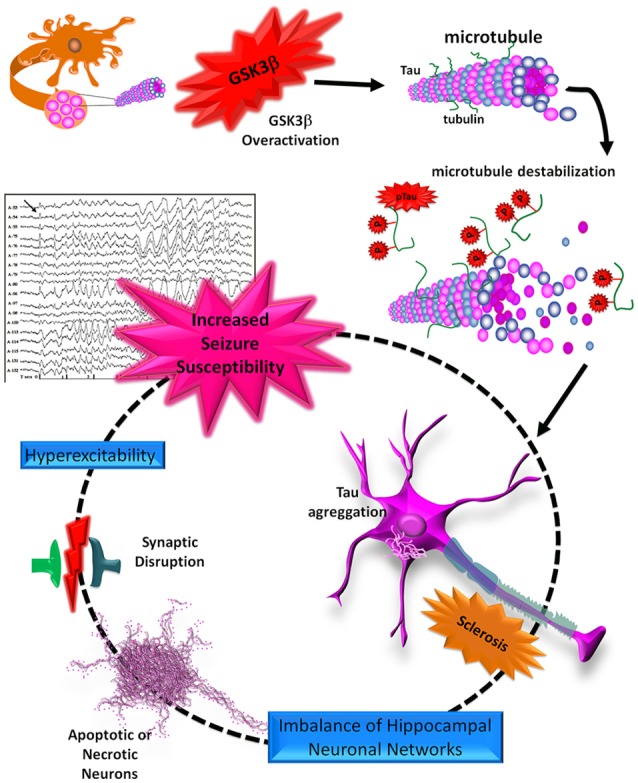
Schematic representation of the roles of tau and GSK3β in the neurodegeneration process. The tau protein can be hyperphosphorylated by an increase in GSK3β activity. pTau loses its union to the MTs, favoring its aggregation and the formation of neurofibrillary tangles (NFTs). These aggregates, like neuritic plaques (NPs), promote an imbalance of hippocampal neuronal networks and synaptic dysfunction, which in turn may favor neuronal death through apoptosis or necrosis. Finally, these alterations lead to processes of hyperexcitability and excitotoxicity in the hippocampus, favoring increased seizure susceptibility.

TLE is a common disorder characterized by hippocampal sclerosis and chronic seizures that are frequently resistant to pharmacological treatment and are associated with disabling comorbidities and cognitive decline. Epidemiological studies have shown an increased prevalence of several dementias, including AD, in chronic epilepsy. These pathologies converge in the hyperphosphorylation of tau protein, apparently mediated by an increased activity of GSK3β that can promote apoptosis, aberrant neurogenesis, impaired synaptic plasticity, and hyperexcitability in the hippocampus. These alterations have been well described in AD, but are not yet completely understood in TLE (Figure 4). For this reason, further studies are necessary to clarify the roles of tau and GSK3β in physiopathology and structural abnormalities and to probe the effectiveness of novel tau immunotherapies. Also, considering that AD and TLE are sporadic and multifactorial diseases, it would be interesting to study different alterations in the GSK3β and the tau (*MAPT*) encoding genes that could represent genetic risk factors for the development of TLE and AD.

## Author Contributions

DT-R, PP-R, MA-V, and VC-P drafted the manuscript. All authors read and approved the final manuscript.

## Conflict of Interest

The authors declare that the research was conducted in the absence of any commercial or financial relationships that could be construed as a potential conflict of interest.
